# Effect of Shenmai injection on preventing the development of nitroglycerin-induced tolerance in rats

**DOI:** 10.1371/journal.pone.0176777

**Published:** 2017-04-28

**Authors:** Qian Zhou, Yan Sun, Wangxiao Tan, Xiao Liu, Yuchen Qian, Xianghui Ma, Ting Wang, Xiaoying Wang, Xiumei Gao

**Affiliations:** 1 State Key Laboratory of Modern Chinese Medicine, Tianjin University of Traditional Chinese Medicine, Tianjin, China; 2 College of Traditional Chinese Medicine, Tianjin University of Traditional Chinese Medicine, Tianjin, China; Temple University School of Medicine, UNITED STATES

## Abstract

Long-term nitroglycerin (NTG) therapy causes tolerance to its effects attributing to increased oxidative stress and endothelial dysfunction. Shenmai injection (SMI), which is clinically used to treat cardiovascular diseases, consists of two herbal medicines, *Ginseng Rubra* and *Ophiopogonjaponicas*, and is reported to have antioxidant effects. The present study was designed to investigate the potential preventive effects of Shenmai injection on development of nitroglycerin-induced tolerance. The present study involves both in vivo and in vitro experiments to investigate nitroglycerin-induced tolerance. We examined the effect of Shenmai injection on the cardiovascular oxidative stress by measuring the serum levels of malondialdehyde (MDA) and superoxide dismutase (SOD). Endothelial dysfunction was determined by an endothelium-dependent vasorelaxation method in aortic rings and NOS activity. Inhibition of the cGMP/cGK-I signalling pathway was determined from released serum levels of cGMP and the protein expression levels of sGC, cGK-I, PDE1A and P-VASP by western blot. Here, we showed that SMI ameliorated the decrease in AV Peak Vel, the attenuation in the vasodilation response to nitroglycerin and endothelial dysfunction. SMI also reduced the cardiovascular oxidative stress by reducing the release of MDA and increasing the activity of SOD. Shenmai injection further ameliorated inhibition of the cGMP/cGK-I signalling pathway triggered by nitroglycerin-induced tolerance through up-regulating the protein expression of sGC, cGK-I, and P-VASP and down- regulating the proteins expression of PDE1A. In vitro studies showed that Shenmai injection could recover the attenuated vasodilation response to nitroglycerin following incubation (of aortic rings) with nitroglycerin via activating the enzymes of sGC and cGK-I. Therefore, we conclude that Shenmai injection could prevent NTG nitroglycerin-induced tolerance at least in part by decreasing the cardiovascular oxidative stress, meliorating the endothelial dysfunction and ameliorating the inhibition of the cGMP/cGK-I signalling pathway. These findings indicate the potential of Shenmai injection (SMI) as a promising medicine for preventing the development of nitroglycerin-induced tolerance.

## Introduction

Nitroglycerin (NTG) has been used for the treatment of angina pectoris for more than 100 years [1]. Despite the variety of anti-angina drugs that have been introduced, NTG still is one of the most commonly prescribed drugs in the management of coronary atherosclerotic heart diseases and angina pectoris due to its potent vasodilator capacity [2]. NTG induces vasodilation by releasing nitric oxide (NO), which can directly activate soluble guanylate cyclase (sGC), and subsequently increase guanosine 3',5'-cyclic phosphate (cGMP) levels [3–5]. The cGMP in turn activates a cGMP-dependent protein kinase I (cGK-I) mediating vasodilation via phosphorylation of proteins that regulate intracellular Ca^2+^ levels [6]. However, it has been observed that long-term administration of NTG can cause the development of tolerance, which is defined as the loss of effects or the need to increase dosages to maintain the effects [7]. Although the underlying mechanisms of NTG-induced tolerance remain unclear, recent studies have proposed possible mechanism, which is the inhibition of the cGMP/cGK-I signaling pathway, due to decreasing cGMP and cGK-I activity and altered cGMP turnover including increased phosphodiesterase (PDE) and decreased sGC activity [8, 9]. The vasodilator-stimulated phosphoprotein (VASP) phosphorylation and in particular VASP serine239 phosphorylation (P-VASP) has been shown to be a useful monitor for cGK-I activity [10]. In addition, a recent study showed that the level of P-VASP in vascular tissue from NTG-treated animals closely correlates with changes in endothelial function and vascular oxidative stress [11, 12]. More recent studies showed that oxidative stress, due to overproduction of reactive oxygen species (ROS), plays an essential role in the development of NTG tolerance [13]. Increased oxidative stress may lead to endothelial dysfunction. In addition, increased ROS may chemically interact with available NO, decreasing its vasodilation effect and interfering with NTG biotransformation, such as inhibiting the activity of mitochondrial aldehyde dehydrogenase (ALDH-2), sGC, and cGK-I [14–17]. In clinical practice, Carvedilol can prevent or attenuate the development of tolerance by its antioxidant effect [18]. However, no medicine is clinically available to inhibit the development of NTG-induced tolerance effectively. Therefore, we proposed to use combined treatment of an antioxidant medicine and NTG to prevent NTG-induced tolerance.

Shenmai injection(SMI), as a traditional Chinese medicine injection, was approved by China Food and Drug Administration (CFDA) for the treatment of heart failure in 1995 [19], which contains extracts of the traditional Chinese remedies *Ginseng Rubra* (*araliaceae*) and *Ophiopogonjaponicas* (*lilia-ceae*). The multiple active components (including ginsenoside Rg1, ginsenoside Re, ginsenoside Rf, ginsenoside Rc, ginsenoside Rd, ginsenoside Rb1, ginsenoside Rb2, ginsenoside Ro and ophiopogonin D) in SMI has been reported [20]. Recent studies have indicated that SMI improves not only angina pectoris but also the endothelial function [21], which may improve endothelial dysfunction triggered by NTG tolerance. In our preliminary study, we found SMI had antioxidant effect, which could decrease oxidative stress. A recent study also reported that SMI has antioxidant effects by reducing ROS and eliminating the production of oxidizing effect such as MDA [22, 23], which may decreasing the cardiovascular oxidative stress induced by NTG tolerance.

On the basis of this evidence, the present study was designed to investigate the potential effects of SMI on preventing the development of NTG-induced tolerance in rats. We used the NTG-induced tolerance model in rats in vivo for studies and also examined the effects of SMI on oxidative stress, endothelial dysfunction and the inhibition cGMP/cGK-I signalling pathway in rats with NTG-induced tolerance.

## Materials and methods

### Animal

Adult male Sprague-Dawley (SD) rats (240–250 g) were purchased from Beijing HFK Bioscience (Beijing, China). This study was carried out in strict accordance with the recommendations in the Guidance Suggestions for the Care and Use of Laboratory Animals issued by the Ministry of Science and Technology of China. The protocol was approved by the Laboratory Animal Ethics Committee of Tianjin University of Traditional Chinese Medicine (Permit Number: TCM-LAEC2016013).

### Regents and drugs

SMI was purchased from CTQ Pharmaceutical Group Co. Ltd. (Hangzhou, China) according to the guidelines of Good Manufacturing Practice and Good Laboratory Practice. SMI’s major components were determined by high performance liquid chromatography finger print [24]. NTG injection was obtained from Beijing Yimin Pharmaceutical Co. Ltd. (Beijing, China). Carvedilol was obtained from Qilu Pharmaceutical Co. Ltd. (Jinan, China). Isoflurane was obtained from YEERAN Technology Co. Ltd. (Beijing, China); Chloral hydrate was obtained from the Tianjin Kermel Chemical Reagent Co. Ltd., (Tianjin, China); Acetylcholine (Ach) and Norepinephrine (NE) were purchased from Sigma-Aldrich (St Louis, MO, U.S.A.); NaCl, KCl, KH_2_PO_4_, MgSO_4_·7H_2_O, CaCl_2_, NaHCO_3_ and Glucose were obtained from the Tianjin Chemical Reagent Factory (Tianjin, China).

### Nitroglycerin tolerance model and drug administration

The rats were randomly divided into six groups: control group (Con), NTG-induced tolerance group (25 mg/kg, Tolerance), co-treatment with low dose SMI (0.4 mL/kg) and NTG group (SMI-L + Tolerance), co-treatment with middle dose SMI (0.8 mL/kg, clinical equivalent dose) and NTG group (SMI-M + Tolerance), co-treatment with high dose SMI (1.6 mL/kg) and NTG (SMI-H + Tolerance) and co-treatment with Carvedilol (4 mg/kg, clinical equivalent dose) and NTG (Carvedilol + Tolerance) group. The rats of co-treatment with SMI and NTG groups were pretreated with SMI via intramuscular injection for seven days; the rats of co-treatment with Carvedilol and NTG were pretreated with Carvedilol via intragastrical administration for seven days. The rats in the control and NTG-tolerance groups were treated with saline. From the eighth day, the rats in all of the groups except the control group were administered 25 mg/kg NTG via subcutaneous injection for seven days. The other groups were treated with relevant reagents similarly for the first seven days. Each experimental group included 15 animals.

### Ultrasound echocardiography evaluation in vivo

The cardiac function was evaluated with echocardiography after the last drug administration. All Rats were anesthetized using 3% inhalant isoflurane in 100% oxygen and maintained in 1.5–2% isoflurane during the echocardiogram testing. M-mode, B-mode and left ventricular outflow tract (LVOT) ultrasound images were obtained by a Vevo 2100 ultrasound system (Visual Sonics, Toronto, Canada). The following parameters including left ventricular ejection fraction percentage (EF %), fractional shortening percentage (FS %) and Aortic flow peak velocity (AV Peak Vel, mm/s) were measured as indicators of the left ventricular function.

### Effect of SMI on endothelium dysfunction induced by Nitroglycerin tolerance

After the last drug administration, rats were anesthetized with 5% chloral hydrate (6 mL/kg) by intraperitoneal injection, and the thoracic aorta was immediately isolated and transferred to petri dishes containing Krebs Henseleit (K-H) (NaCl118 mM, KCl 4.75 mM, MgSO_4_·7H_2_O 1.2 mM, KH_2_PO_4_ 1.2 mM, CaCl_2_ 2.5 mM, NaHCO_3_ 25 mM and glucose 11 mM). The surrounding fat and connective tissues of thoracic aorta were removed, and the trimmed aorta was subsequently cut into 3- to 5-mm rings cautiously to avoid any inadvertent endothelial damage. The rings were suspended horizontally between two parallel stainless hooks. One fixed to the bottom of the organ bath filled with warmed (37°C) and oxygenated (95% O_2_ and 5% CO_2_) K-H solution, while the other connected to a force displacement transducer (AD Instruments Pty Ltd., Australia). A computer-assisted data acquisition system (PowerLab/4SP; AD Instruments, Australia) recorded the changes in isometric tension during the experiments [25]. The aorta rings were equilibrated at a based tension of 2.0 g for 60 minutes, and the K-H solution was changed every 15 minutes. Every experiment started with a repeated KCl treatment to test the contractility, after which the rings were rinsed with a pre-warmed and oxygenated K-H solution until the muscle tension returned to the basal level [26]. The endothelial function was evaluated by NE and Ach. First, 10^−6^ M NE was added to achieve a plateau phase, and 10^−5^ M of Ach was later added to induce vasoconstriction. The aortic rings were washed 3 times in 15-minute intervals to remove NE and Ach completely. The aortic rings were precontracted with NE (10^−6^ M), and then incubated with incremental doses of NTG (10^−9^–10^−4^ M) in 5-minute intervals. The primary concentration–response curve to NTG was obtained.

At the end of the experiments (drawing blood from the abdominal aorta), the serum was collected and centrifuged at 3000 rpm for 10 minutes storing at -80°C.We investigated the activity of nitric oxide synthase (NOS), including total NOS, endothelial NOS (eNOS) neuronal NOS (nNOS) and inducible NOS (iNOS) by NOS typed assay kit, eNOS Elisa Assay Kit and nNOS Elisa Assay Kit (Nanjing Jiancheng Bioengineering Institute, Nanjing, China). The content of NO was investigated by the NO Assay Kit (Beyotime Institute of Biotechnology, Jiangsu, China). All of the procedures were performed according to the manufacturer’s instructions, and the light absorbance was detected by an Enspire multimode plate reader (Perkin Elmer, America).

### Effect of SMI on oxidative stress induced by Nitroglycerin tolerance

The serum levels of reactive oxygen species (ROS), lipid peroxidation product malondialdehyde (MDA) and superoxide dismutase (SOD) were determined to evaluate the oxidative stress triggered by NTG-induced tolerance and the antioxidant activity of SMI. In addition, hydrogen peroxide (H_2_O_2_), catalase (CAT) and glutathione peroxidase (GSH-PX) also were determined to evaluate the oxidative stress. The content of ROS, MDA and H_2_O_2_ were measured by rat ROS ELISA Kit (TSZ Biosciences, America), MDA Assay Kit (Nanjing Jiancheng Bioengineering Institute, Nanjing, China) and H_2_O_2_ Assay Kit (Nanjing Jiancheng Bioengineering Institute, Nanjing, China). The activity of SOD, CAT and GSH-PX were separately investigated by SOD Assay Kit (Nanjing Jiancheng Bioengineering Institute, Nanjing, China), CAT Assay Kit (Nanjing Jiancheng Bioengineering Institute, Nanjing, China) and GSH-PX Assay Kit (Nanjing Jiancheng Bioengineering Institute, Nanjing, China). All of the procedures were performed according to the manufacturer’s instructions. The light absorbance was detected by an Enspire multimode plate reader (Perkin Elmer, America).

### Effect of SMI on the cGMP/cGK-I signalling pathway

The serum levels of cGMP were measured by cGMP Direct Immunoassay Kit (BioVision, San Francisco, U.S.A.) according to the manufacturer’s instruction, and the light absorbance was detected by an Enspire multimode plate reader (Perkin Elmer, America).

Isolated aortic tissue was frozen and homogenized in liquid nitrogen. The protein concentrations were determined using a BCA protein assay kit (Thermo Scientific, MA, USA). The sample volumes were adjusted to normalize the total protein concentration Standard Western blot analysis was performed, as previously described [27]. Proteins were separated by SDS-Page and blotted onto PVDF membranes. After blocking, immunoblotting was performed with the following antibodies: polyclonal rabbit Anti–GADPH (36 kDa,1:1000, Millipore, USA) as acontrol for loading and transfer, polyclonal rabbit Anti-sGCα1 (80 kDa, 1:10000, Abcam, UK), polyclonal rabbit Anti-sGCβ1 (71 kDa, 1:5000, Abcam, UK), polyclonal rabbit Anti-PDE1A (61 kDa, 1:5000, Abcam, UK), polyclonal rabbit Anti- VASP (42 kDa, 1:1000, Abcam, UK) and polyclonal rabbit Anti-P-VASP (phosphor S157, 40 kDa, 1:1000, Abcam, UK), polyclonal rabbit Anti- cGK-I (78 kDa, 1:1000, Abcam, UK). The protein bands were detected by chemiluminescence (ECL, Thermo Scientific, MA, USA), and densitometric analyses were performed using Scion Image software (Scion Corp, Frederick, MD, USA).

### Effect of SMI on Nitroglycerin tolerance in rat aortic rings

Preparation of isolated thoracic aortic rings of rats (new, 240–250 g) as previously describe [25]. Confirmed the intactness of endothelium when a significant relaxation (more than 80%) occurred in response to Ach (10^−5^ M) in the rings precontracted with NE (10^−6^ M). Induction of NTG Tolerance in Rat Thoracic Aorta was achieved as follows.

Phase 1: After confirming the intactness of endothelium, the aortic rings were precontracted with NE (10^−6^ M) and were incubated with incremental doses of NTG (10^−9^–10^−4^ M) in 5-minute intervals. The primary concentration–response curve to NTG was obtained.
Phase 2: The aortic rings were washed 3 times in 15-minute intervals to remove NE and NTG completely. Next, the rings were incubated with NTG (10^−5^ M) for 1 hour.
Phase 3: The aortic rings were washed 3 times in 10-minute intervals to clear out NTG completely.
Phase 4: The secondary concentration–response curve to NTG was obtained after a constant contraction to NE was achieved.

To preclude the possibility of spontaneous attenuation of the contraction by time, some of the aortic rings were bathed in NTG-free Krebs solution for 1 hour, and their secondary concentration–response curve to NTG was obtained.

To investigate the effect of SMI on NTG-induced tolerance, all above the phases 1–4 were repeated. The only difference was added different concentrations of SMI (2.5, 5.0, 10 μL/mL) in phase 2 along with NTG (10^−5^ M).

### Effect of sGC activator and cGK activator on Nitroglycerin tolerance in rat aortic rings

All the above phases 1–4 were repeated in this set of experiment. The only difference was adding either sGC activator (BAY41-2272, 10^−5^ M) or cGK activator (8-Br- cGMP, 10^−5^ M) in phase 2 along with NTG (10^−5^ M).

### Effect of SMI on the activity of sGC and cGK-I in rat aortic rings

The above phases 1, 3, 4 were repeated in this set of experiment. In phases 2, the aortic rings were washed 3 times in 15-minute intervals to remove norepinephrine and NTG completely. Next, different concentrations of SMI (2.5, 5.0, 10 μL/mL) were added ten minutes later, and they were incubated with inhibitor of sGC (ODQ, 10^−5^ M) or inhibitor of cGK-I (KT5823, 10^−5^ M) for 30 minute.

To preclude the possibility of spontaneous attenuation of the contraction by time, some of the aortic rings were bathed in ODQ-free or KT5823-free K-H solution for 30 minute, and their secondary concentration–response curve to NTG was obtained.

### Statistical analysis

All of the data are expressed as the means ± standard error mean (SEM). SPSS 17.0 was used for statistical analysis of the data. Statistical significance was assessed by one-way analysis of variance (ANOVA) followed by Tukey’s test. Values of *P*<0.05 were considered to be statistically significant.

## Results

### Echocardiography evaluation

**Fig 1:**
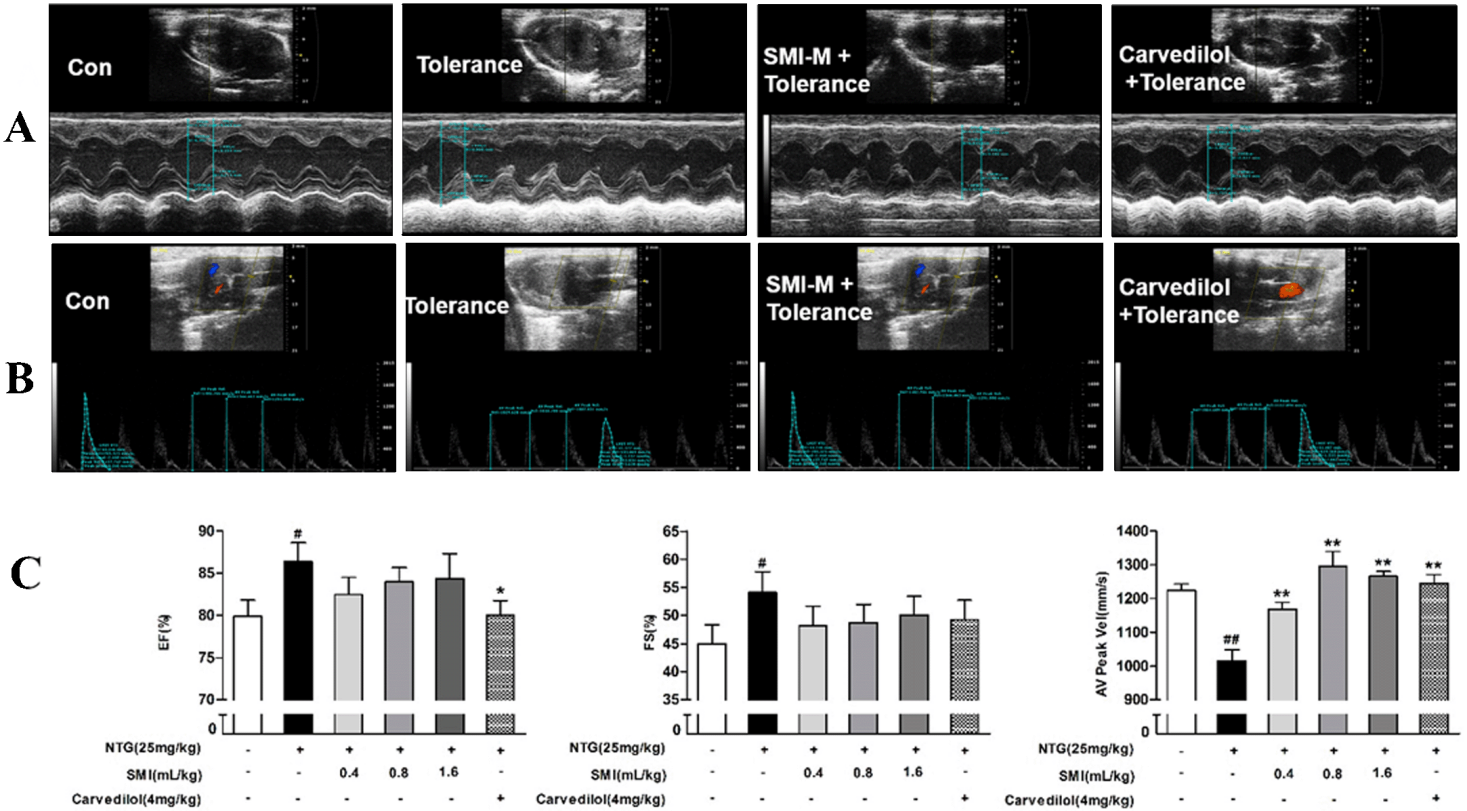
Co-treatment with SMI and NTG protected the cardiac function in vivo. (A) Representative M-mode echocardiograms of control, the NTG-induced tolerance group and co-treatment groups showed the wall motion. **(B)** AV Peak Vel echocardiograms on Doppler of the left ventricular outflow tract. **(C)** EF, FS and AV peak Vel in the groups. Data are expressed as the mean ± SEM; n = 9 in each group; ^#^P < 0.05 versus the control group, *P < 0.05 versus the NTG-induced tolerance group.

We confirmed the effect of co-treatment with SMI and NTG on NTG-induced tolerance by quantitative analysis of the echocardiograms. The representative echocardiograms in controls, NTG-induced tolerance group, co-treatment group are presented in Fig 1. The rats were treated with NTG (25 mg/kg) or a co-treated with NTG and SMI (0.4, 0.8, 1.6 mL/kg), and examined by M-mode and LVOT echocardiography (Fig 1A and 1B). As shown in Fig 1C, compared with the control group, the EF and FS were significantly increased in the rats treated with NTG (*P<*0.01), but the AV peak Vel was significantly decreased in the rats treated with NTG (*P<*0.01). Co-treatment with either SMI (0.4, 0.8, 1.6 mL/kg) or Carvedilol and NTG significantly increased the AV Peak Vel (*P<*0.05) whereas co-treatment with Carvedilol and NTG significantly decreased the EF (*P<*0.05), but did not change FS. Co-treatment with SMI and NTG showed a trend of decreasing the EF and FS, but it was not significant.

### SMI prevents Nitroglycerin tolerance in vivo

**Fig 2:**
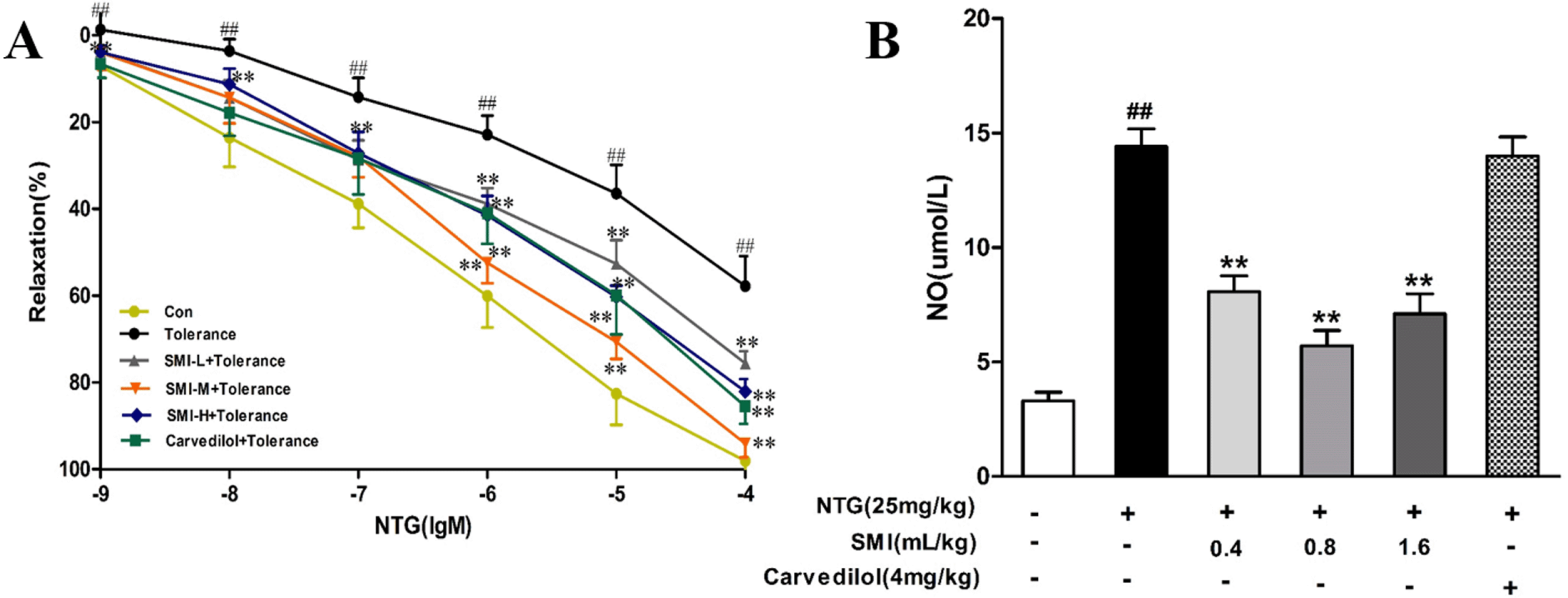
Confirmation of NTG-induced tolerance. (A) Vascular relaxation induced by NTG by NE-induced vasoconstriction. **(B)** Detection of the release of NO. The data are expressed as the mean ± SEM; n = 9 in each group; ^##^P < 0.01 versus control, **P < 0.01 versus the NTG-induced tolerance group.

A marked attenuation of vasodilation effect of NTG by reducing the release of NO is a characteristic feature of NTG-induced tolerance. We confirmed the NTG-induced tolerance by investigating vasodilation of NTG using the aortic ring assay, and detected the release of NO by the Nitric Oxide Assay Kit. As shown in Fig 2A, compared with the control group, the concentration-response curve of NTG in rat (treated with NTG) aortic rings significantly moved up (*P<*0.01), indicating that the vasodilation effect to NTG was significantly weakened in rats treated with NTG. Co-treatment with either SMI (0.4, 0.8, 1.6 mL/kg) or Carvedilol significantly moved down the concentration-response curve of NTG (*P<*0.01), indicating that the vasodilation effect to NTG was significantly enhanced in rats which received co-treatment with SMI and NTG. As shown in Fig 2B, compared with the control group, the release of NO was significantly increased in rats treated with NTG (*P<*0.01). Co-treatment with SMI (0.4, 0.8, 1.6 mL/kg) significantly decreased the release of NO up to the normal level (*P<*0.01).

### SMI improves endothelium dysfunction induced by Nitroglycerin tolerance

**Fig 3:**
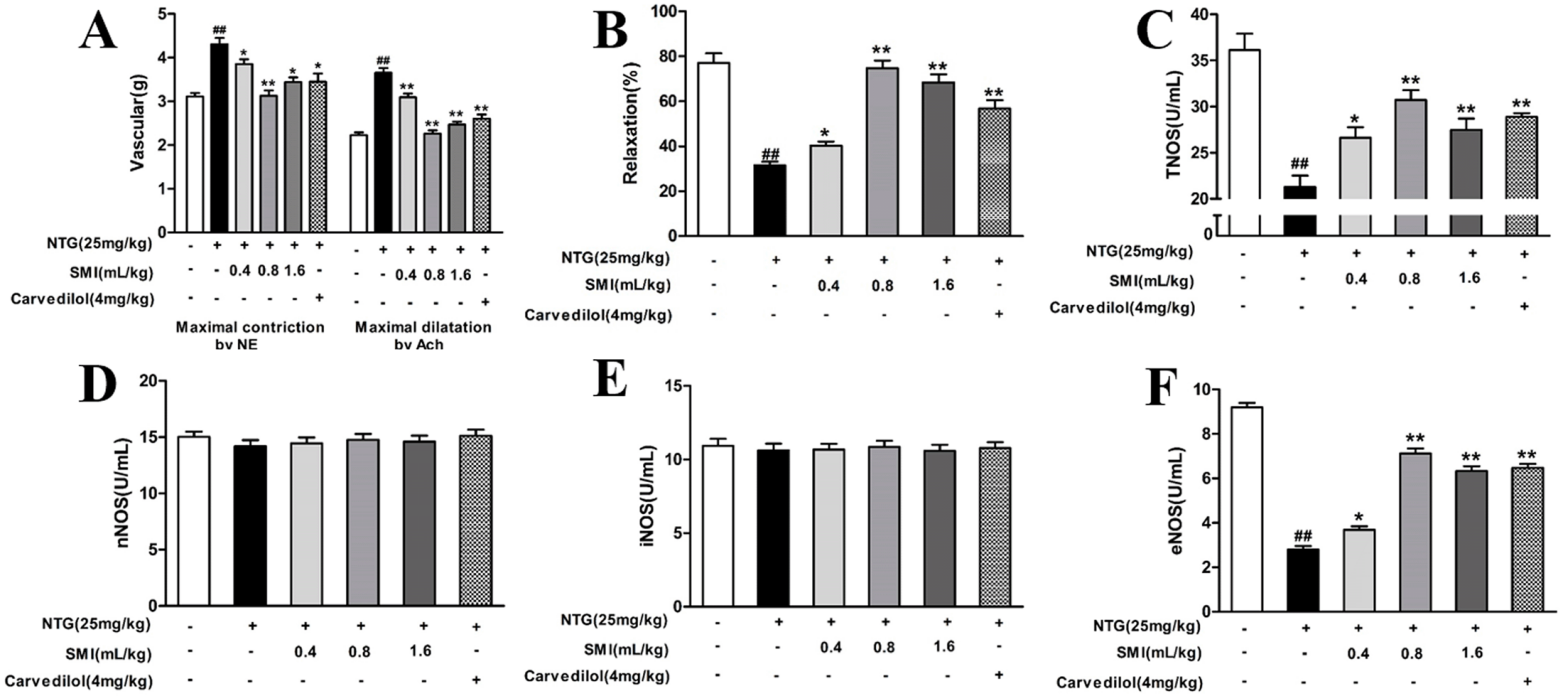
SMI reduced endothelial dysfunction of NTG-induced tolerance. (A) Maximal constriction and dilation by NE and Ach. **(B)** The maximal vasodilatation of Ach by NE-induced constriction. **(C)** Detection of the activity of TNOS. **(D)** Detection of the activity of nNOS. **(E)** Detection of the activity of iNOS. **(F)** Detection of the activity of nNOS. The data are expressed as the mean ± SEM; n = 9 in each group; ^#^P < 0.05 versus control, *P < 0.05 versus the NTG-induced tolerance group; ^##^P < 0.01 versus control, **P < 0.01 versus the NTG-induced tolerance group.

The aorta rings were pre-treated with 1 μM NE to achieve a plateau phase. In the control group, the vascular tone of aorta rings reached 3.11 ± 0.23 g from the baseline value of 2.0 g. Next, 10 μM Ach was added to induce vasodilation, the vascular tone of aorta rings reached 2.227 ± 0.12 g, and the maximal vasodilation was 79%. In the NTG treated rats, the maximal constriction by NE was significantly increased to 4.31 ± 0.41 g. With the Ach administration, the vascular tone was reduced to 3.58 ± 0.30 g, and the maximal vasodilatation was 32%, indicating significant endothelial dysfunction. Compared with the NTG treated group, the groups co-treated with either SMI or Carvedilol showed significantly reduced NE-induced constriction and more Ach-induced relaxation, and the maximal vasodilatation was significantly increased (Fig 3A and 3B). Compared with the control group, the activity of TNOS was significantly decreased in rats treated with NTG (*P<*0.01). Co-treatment with either SMI or Carvedilol significantly enhanced the activity of TNOS (*P<*0.05); but in three types of NOS, only the activity of eNOS was significantly decreased in rats treated with NTG (*P<*0.01), Co-treatment with either SMI or Carvedilol significantly augmented the activity of eNOS (*P<*0.05, *P<*0.01) (Fig 3C–3F).

### SMI reduces oxidative stress induced by Nitroglycerin tolerance

**Fig 4:**
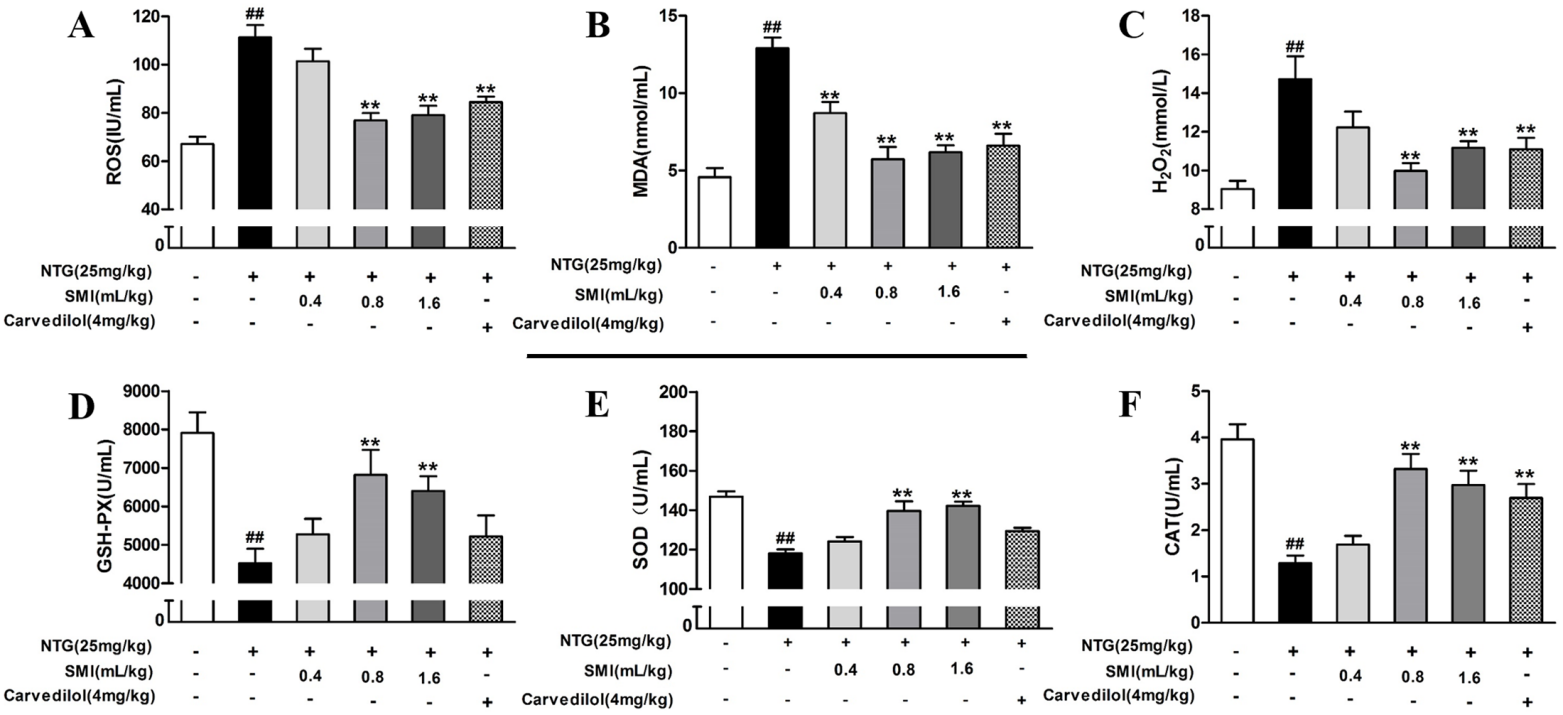
SMI reduced oxidative stress of NTG-induced tolerance. (A) Detection of ROS production. **(B)** Detection of MDA production. **(C)** Detection of H2O2 production. **(D)** Detection of GSH-PX activity. **(E)** Detection of SOD activity. (F) Detection of CAT activity. The data are expressed as the mean ± SEM; n = 9 in each group; ^##^P < 0.01 versus control, **P < 0.01 versus the NTG -induced tolerance group.

Oxidative stress was measured by detecting the production of ROS, MDA and H_2_O_2_ and the activity of SOD, CAT and GSH-PX. As shown in Fig 4, compared with the control group, the release of ROS, MDA and H_2_O_2_ were significantly increased in the rats treated with NTG (*P<*0.01), and the activity of SOD, CAT and GSH-PX were significantly decreased (*P<*0.01), indicating that rats treated with NTG could develop oxidative stress. Co-treatment with either SMI or Carvedilol significantly decreased the production of ROS, MDA and H_2_O_2_ (*P<*0.01) (Fig 4A–4C), and increased the activity of GSH-PX, SOD and CAT (*P<*0.01) (Fig 4D–4F).

### SMI improves inhibition of the cGMP/cGK-I signalling pathway occurring due to Nitroglycerin tolerance

**Fig 5:**
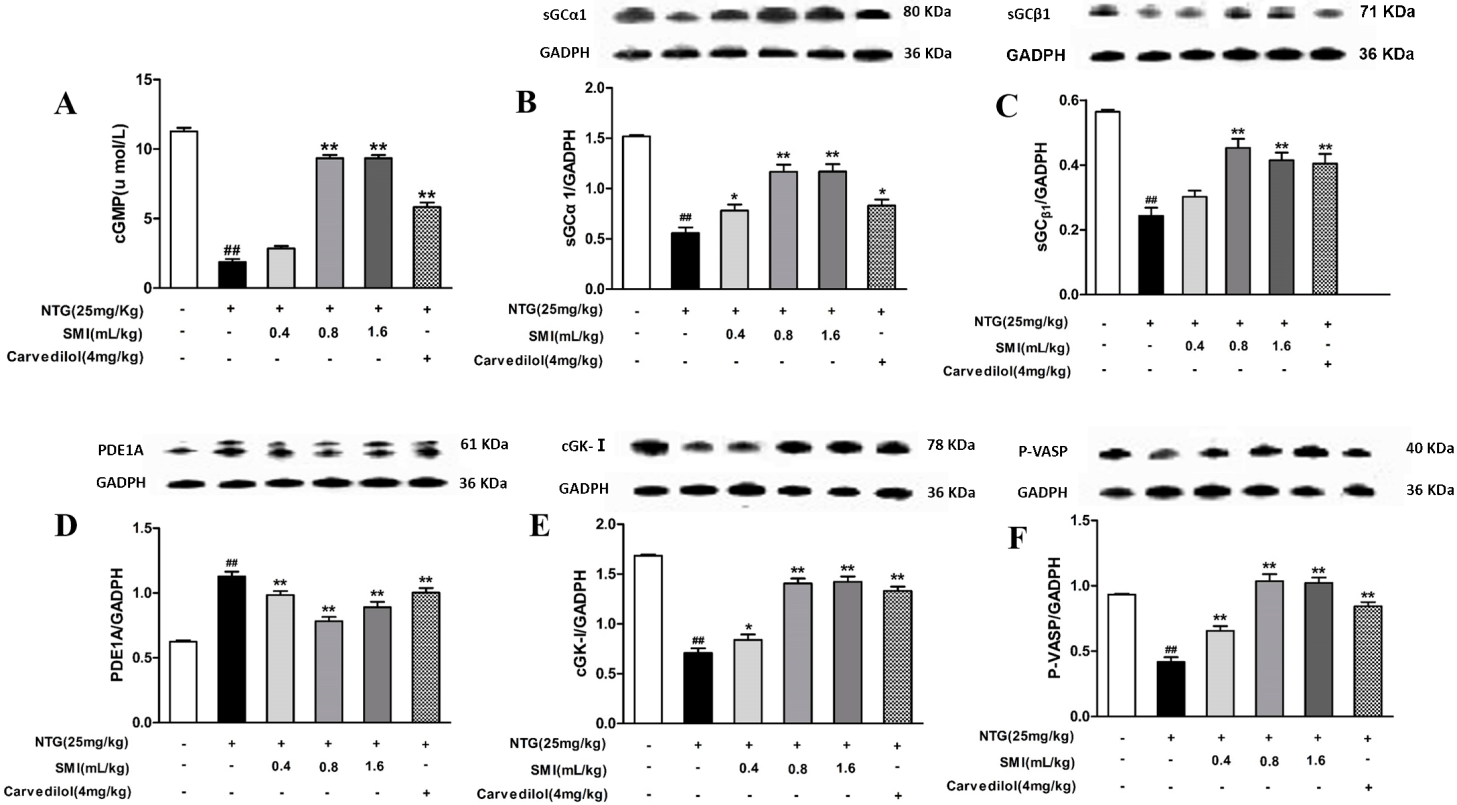
SMI improved the injury of the cGMP/cGK-I signalling pathway occurring due to NTG-induced tolerance. (A) Detection of the release of cGMP. **(B)** Protein expression of sGCα1. **(C)** Protein expression of sGCβ1. **(D)** Protein expression of PDE1A. **(E)** Protein expression of cGK-I. **(F)** Protein expression of serin239 phosphorylated VASP (P-VASP). The data are expressed as the mean ± SEM; n = 9 in each group; ^#^P < 0.05 versus control, *P < 0.05 versus the NTG-induced tolerance group; ^##^P < 0.01 versus control, **P < 0.01 versus the NTG-induced tolerance group.

Fig 5A shows the release of cGMP significantly decreased in rats treated with NTG (*P<*0.01). Co-treatment with either SMI (0.8, 1.6 mL/kg) or Carvedilol significantly increased the release of cGMP (*P<*0.01). As shown in Fig 5B to 5F, compared with the control group, the NTG treatment led to a significant decrease in protein expression of the sGCα1, sGCβ1, cGK-I and P-VASP (*P*< 0.01), and a significant increase in protein expression of the PDE1A. Co-treatment with either SMI (0.4, 0.8, 1.6 mL/kg) or Carvedilol significantly increased the expression of sGCα1, cGK-I and P-VASP (*P*< 0.05, *P*< 0.01). Co-treatment with SMI (0.8, 1.6 mL/kg) significantly increased the expression of sGCβ1 (*P*< 0.01).

### SMI improve Nitroglycerin tolerance in rat aortic rings

**Fig 6:**
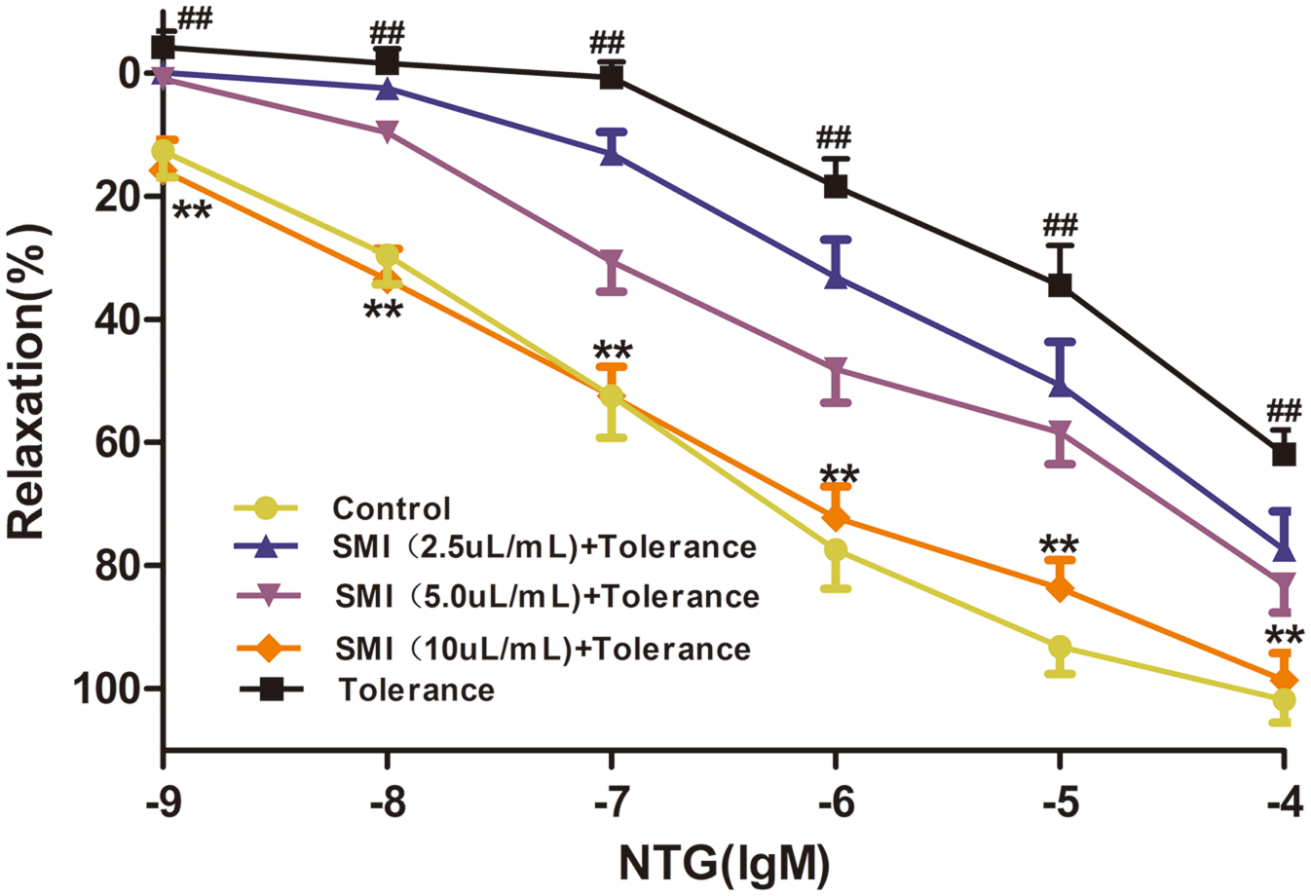
SMI reduced NTG-induced tolerance in rat aortic rings. The data are expressed as the mean ± SEM; n = 15 in each group; ^##^P < 0.01 versus control, **P < 0.01 versus the NTG-induced tolerance group.

As shown in Fig 6, compared with the control vessels, the concentration-response curve of NTG treatment of aortic rings following 1 h incubation (10^−5^ M) for 1 h significantly moved up (*P<*0.01), indicating the vasodilation effect of NTG was significantly attenuated in aortic rings. When co- incubated with SMI (10 μL/mL), the concentration-response cure of NTG significantly moved down (*P<*0.01), indicating that the vasodilation effect of NTG was significantly enhanced.

### Activator of sGC and cGK-I reduce Nitroglycerin tolerance in rat aortic rings

**Fig 7:**
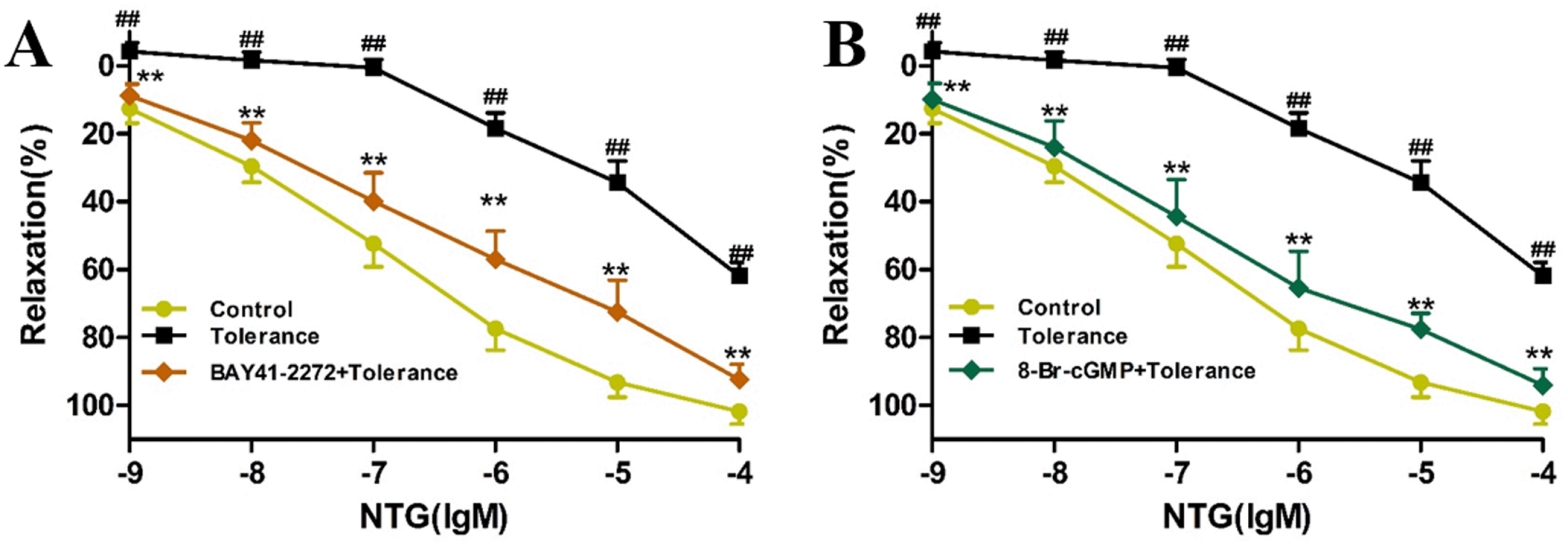
The activator of sGC and cGK-I reduce NTG-induced tolerance in rat aortic rings. **(A)** The activator of sGC reduce NTG-induced tolerance in rat aortic rings. **(B)** The activator of cGK-I reduce NTG-induced tolerance in rat aortic rings. The data are expressed as the mean ± SEM; n = 9 in each group; ^##^P < 0.01 versus control, **P < 0.01 versus the NTG-induced tolerance group.

According to Fig 7, compared with the control vessels, the concentration-response curve of NTG, after aortic rings were incubated with NTG (10^−5^ M) for 1 h, significantly moved up (*P<*0.01), indicating the vasodilation effect of NTG significantly attenuated in aortic rings. Co- incubation with either sGC activation (BAY41-2272, 10^−5^ M) or cGK-I activation (8-Br-cGMP, 10^−5^ M), significantly moved down the concentration-response cure of NTG (*P<*0.01).

### SMI promotes the activity of sGC and cGK-I in rat aortic rings

**Fig 8:**
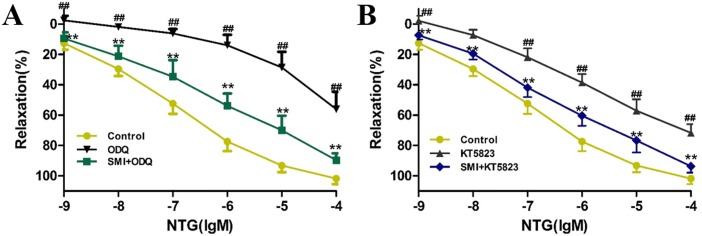
SMI improved the activity of sGC and cGK-I in rat aortic rings. **(A)** SMI improved the activity of sGC in rat aortic rings. **(B)** SMI improved the activity of cGK-I in rat aortic rings. The data are expressed as the mean ± SEM; n = 9 in each group; ^##^P < 0.01 versus control, **P < 0.01 versus the ODQ or KT5823 induced vasorelaxation dyfunction group.

In Fig 8, compared with the control vessels, the concentration-response curve of NTG after aortic rings were incubated with either the inhibitor of sGC (ODQ, 10^−5^ M) or inhibitor of cGK-I (KT5823, 10^−5^ M) for 30 minutes significantly moved up (*P<*0.01), indicating that the vasodilation effect of NTG was significantly attenuated in aortic rings. Co-incubation with SMI (10 μL/mL) significantly moved down the concentration-response curve to NTG (*P<*0.01), indicating that the vasodilation effect of NTG significantly enhanced.

## Discussion

Several clinical and basic studies have shown that long-term treatment with NTG causes the development of tolerance [28]. A previous study reported that long-term treatment with NTG could lead to a marked attenuation of vasodilation response to NTG as observed in the aortic ring assay [29]. Using this approach, we also showed the vasodilation effect of NTG was significantly attenuated in rats treated with NTG. We also showed that co-treatment with SMI could significantly enhance the vasodilation effect of NTG. In addition, via evaluation of echocardiography, we confirmed that co-treatment with SMI could improve the decrease in the AV peak Vel of LVOT in rats triggered by NTG-induced tolerance. It is well-known that NTG exerts vasodilation effect by the release of NO or NO-related species [30]. Recent evidence from an animal study showed a significant reduction in the release of NO in rats which developed NTG-induced tolerance [31]. However, in the present study, the release of NO in rats with NTG-induced tolerance was significantly increased. Nevertheless, the release of NO was restored to normal levels by co-treatment with SMI. The increased release of NO from NTG-induced tolerance may account for oxidative inactivation of NO.

It has been demonstrated that the increased cardiovascular oxidative stress is closely related to NTG-induced tolerance [32]. It was shown that the oxidative stress played a pivotal role in the development of NTG-induced tolerance, which was triggered by overproduction of reactive oxygen species (ROS) [33]. The effect of SMI as an antioxidant and removal of oxygen free radicals and ROS has been reported [34]. As our results noted, co-treatment with SMI and NTG can significantly decrease the release of ROS, MDA and H_2_O_2_, and increase the activity of GSH-PX, SOD and CAT, indicating that SMI reduces cardiovascular oxidative stress evoked by NTG-induced tolerance.

Furthermore, it has been suggested that NTG-induced tolerance is associated with incremental endothelial dysfunction [35]. The direct evidence for this conclusion stems from the finding which showed that endothelial dysfunction occurred with decrease in the activity of eNOS [36].In the current study, we demonstrated that co-treatment with SMI significantly increased the activity of eNOS. In addition, we confirmed that NTG-induced tolerance results in significant endothelial dysfunction though endothelium-dependent vasorelaxation in the aortic ring. According to a previous study, the relaxation to Ach induced constriction by NE was more than 80% compared with the plateau phase in the endothelium-intact aortas and less than 30% in the endothelium-denuded aortas [37]. In our results, the vasodilation response to Ach of the rats treated with NTG attenuated to 31%, and if co-treated with SMI, enhanced to 75%, indicating that SMI significantly improved the endothelial dysfunction triggered by NTG-induced tolerance. The reason why SMI could improve the endothelial dysfunction triggered by NTG-induced tolerance may be due to the protective effect of SMI on endothelial cells, as previous study proposed [38].

It has been suggested that the cGMP appears to be a key player in NTG-induced vasodilation by reducing intracellular Ca^2+^, which is regulated by activating sGC [39]. In addition, the degradation of cGMP is catalysed by several differentially expressed PDE families, especially PDE1A. Therefore, there are two possible mechanisms for the development of NTG-induced tolerance, namely, decreased activity of the sGC and increased the activity of the PDEs, both of which decrease the cGMP level and subsequently attenuate the vasodilation effect of NTG [40, 41]. The first direct evidence for NTG-induced protein expression of PDEs contributing to NTG tolerance dates back to a previous study, which reported that cGMP levels are significantly reduced in rats with NTG-induced tolerance [40]. More recent evidence using rat thoracic aorta shows that long-term treatment with NTG leads to protein expression of sGC (consisting of sGCα1 and sGCβ1protein subunits), which significantly decrease along with a significant reduction of cGMP [42]. It is worth noting that as the cGMP levels reduce, the vasodilation effects significantly vanish in rats with NTG-induced tolerance, as noted by previous studies [43–45]. In addition, Berdeaux et al indicated that exposure of human umbilical vein endothelial cells (HUVEC) to NTG for 16 h led to a significant decrease in cGMP levels [44]. In the present study, we demonstrated that SMI could meliorate the reduced cGMP level triggered by NTG-induced tolerance though up-regulating the protein expression of sGCα1 and sGCβ1 and down-regulating the proteins expression of PDE1A. In our previous study, we showed that long-term treatment with NTG resulted in a significantly decreased protein expression of cGK-I in rat thoracic aorta [12]. In addition, Knorr, M.et al have shown that the activity of phosphorylation of P-VASP at serine 239 (P-VASP) was significantly decreased in rats with chronic NTG treatment [46], which reflected the functional integrity of the NO/cGMP/ cGK-I signalling pathway in vascular tissue was impaired. Fortunately, this findings was in line with our present study where we also discovered that co-treatment with SMI could significantly increase the proteins expression of cGK-I and P-VASP. This finding indicated that SMI could prevent the development of NTG-induced tolerance by reversing the impairment of the NO/cGMP/cGK-I signalling pathway.

On the basis of these findings, we proposed a hypothesis that SMI may activate cGC and cGK. To test this hypothesis, we conducted a series of aortic ring assays. In a previous study, we had demonstrated that activation of sGC or cGK could significantly enhance the vasodilation response to NTG in rats aortic rings with NTG-induced tolerance [47]. Furthermore, we found that pretreatment with SMI could significantly enhance the attenuated vasodilation response to NTG induced by prolonged incubation with the inhibitors of sGC (ODQ) and cGK (KT5823). Therefore, this study is the first to demonstrate that SMI may cause activation of cGC and cGK.

## Conclusion

The present study showed that SMI could prevent NTG-induced tolerance by decreasing the cardiovascular oxidative stress, meliorating the endothelial dysfunction and ameliorating the inhibition of the cGMP/cGK-I signalling pathway. These findings indicate the potential of SMI as a promising reagent for preventing the development of NTG-induced tolerance in the clinic.

## Supporting information

S1 Fig The expression of sGCα1. The original figure of sGCα1 expression by western-blot.
(TIF)

S2 Fig The expression of GADPH. This is an original figure for GADPH as the internal reference of sGCα1 by western-blot.
(TIF)

S3 Fig The expression of sGCβ1. The original figure of sGCβ1 expression by western-blot.
(TIF)

S4 Fig The expression of GADPH. This is an original figure for GADPH as the internal reference of sGCβ1 by western-blot.
(TIF)

S5 Fig The expression of PDE1A. The original figure of PDE1A expression by western-blot.
(TIF)

S6 Fig The expression of GADPH. This is an original figure for GADPH as the internal reference of PDE1A by western-blot.
(TIF)

S7 Fig The expression of cGK-I. The original figure of cGK-I expression by western-blot.
(TIF)

S8 Fig The expression of GADPH. This is an original figure for GADPH as the internal reference of cGK-I by western-blot.
(TIF)

S9 Fig The expression of P-VASP. The original figure of P-VASP expression by western-blot.
(TIF)

S10 Fig The expression of GADPH. This is an original figure for GADPH as the internal reference of P-VASP by western-blot.
(TIF)

S1 Text Chemical characterization of the Shenmai injection.doc. Ultra-performance liquid chromatography/quadrupole time-of-flight mass spectrometry (UPLC/Q-TOF-MS) analysis of Shenmai injection.
(DOC)
