# Supplementary figures and images for: Effect of Shenmai injection on preventing the development of nitroglycerin-induced tolerance in rats
Source: PLoS One. 2017 Apr 28;12(4):e0176777. doi: 10.1371/journal.pone.0176777 (PMC5409518; doi:10.1371/journal.pone.0176777)

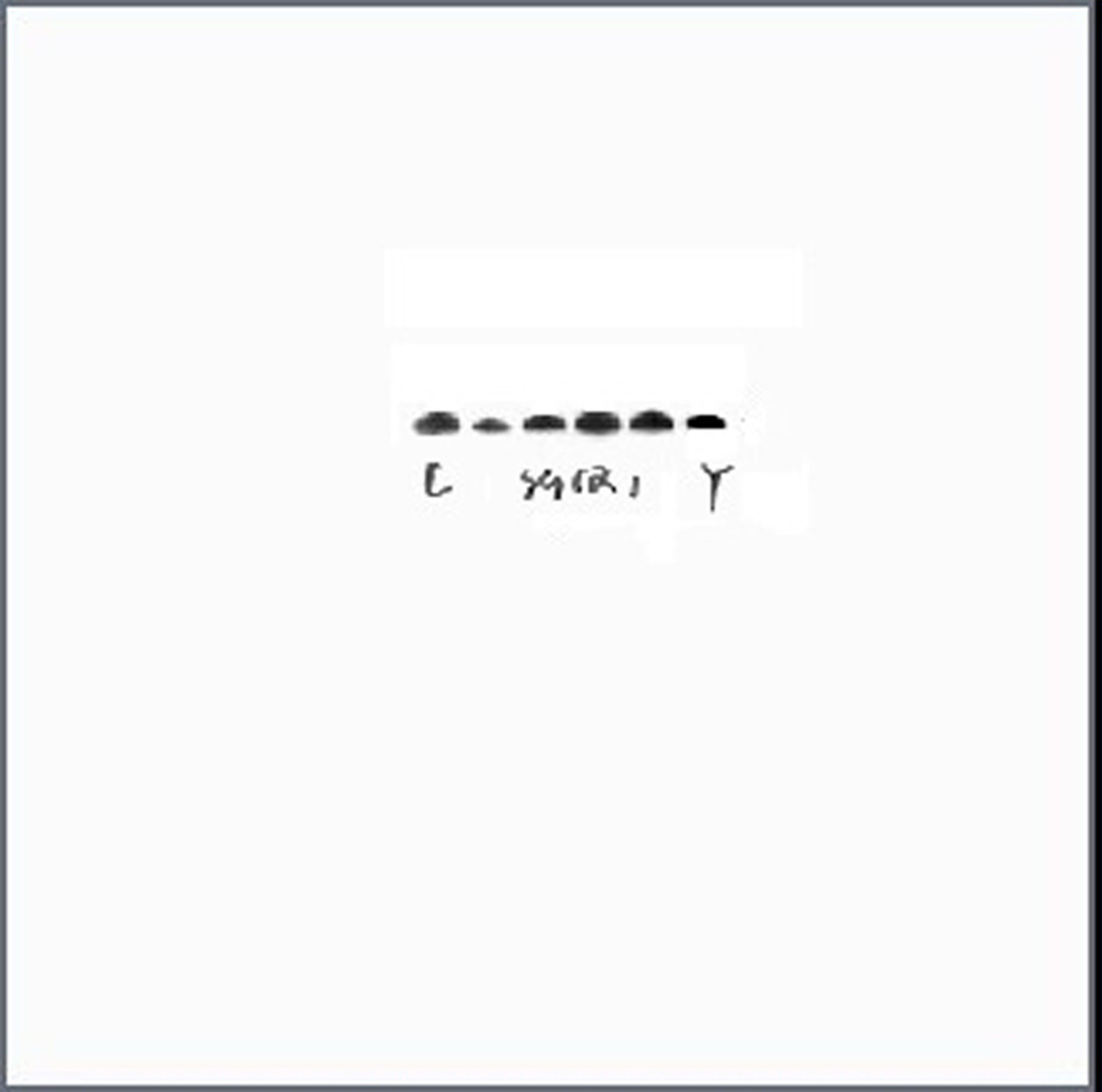

Supplement: S1 Fig — The original figure of sGCα1 expression by western-blot. (TIF) [file pone.0176777.s001.tif]

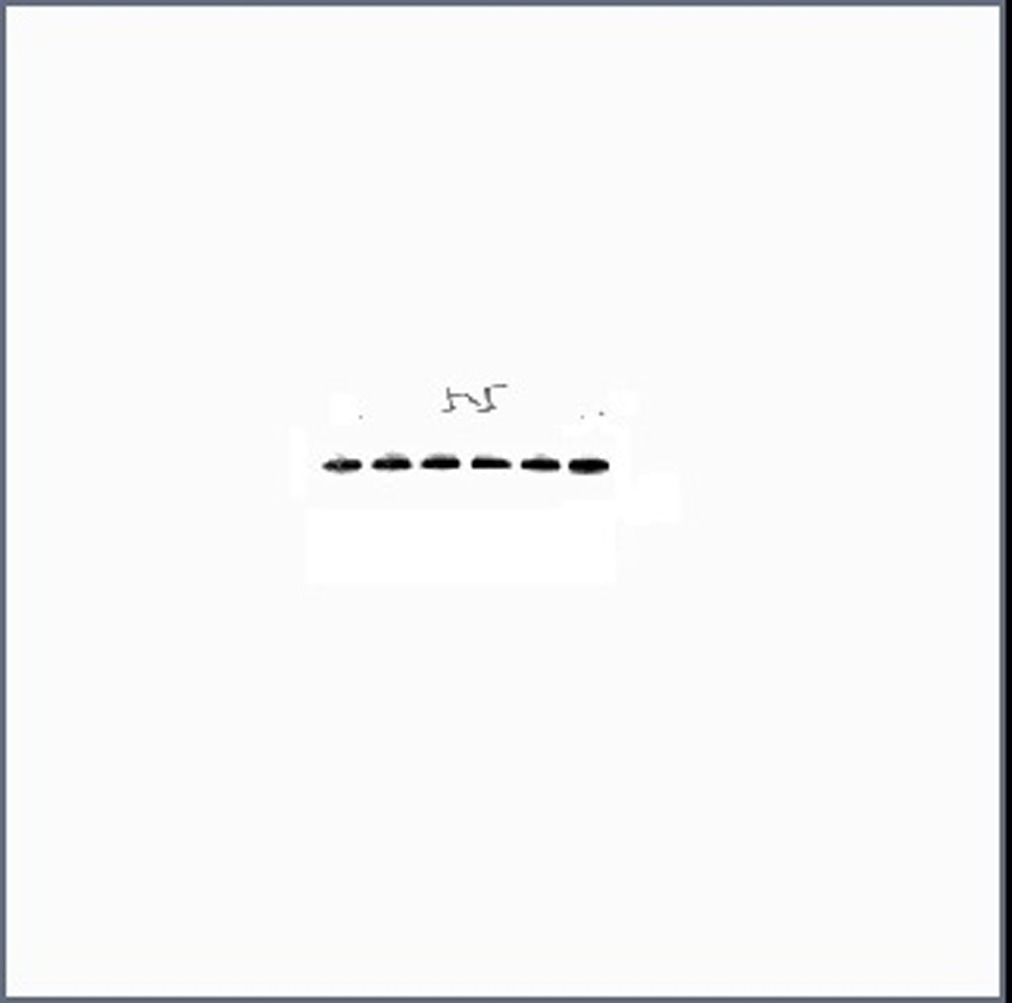

Supplement: S2 Fig — This is an original figure for GADPH as the internal reference of sGCα1 by western-blot. (TIF) [file pone.0176777.s002.tif]

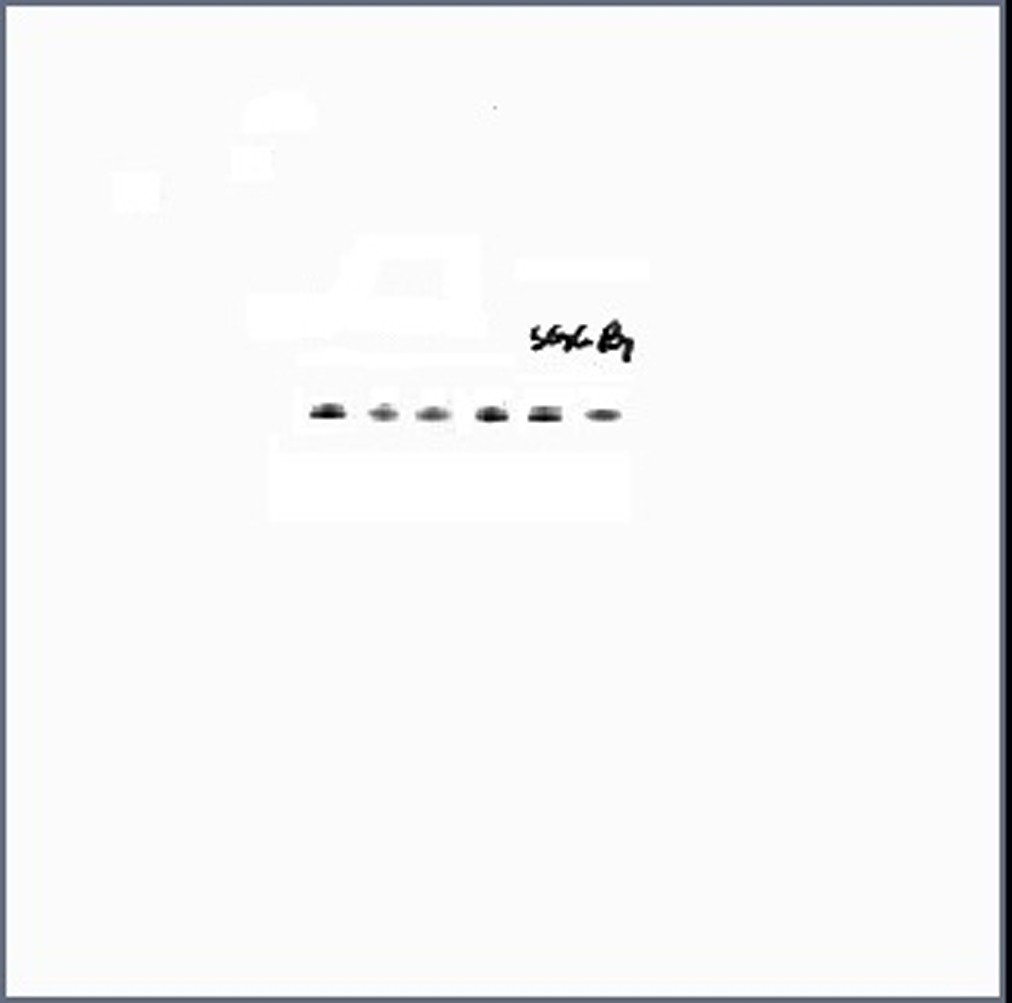

Supplement: S3 Fig — The original figure of sGCβ1 expression by western-blot. (TIF) [file pone.0176777.s003.tif]

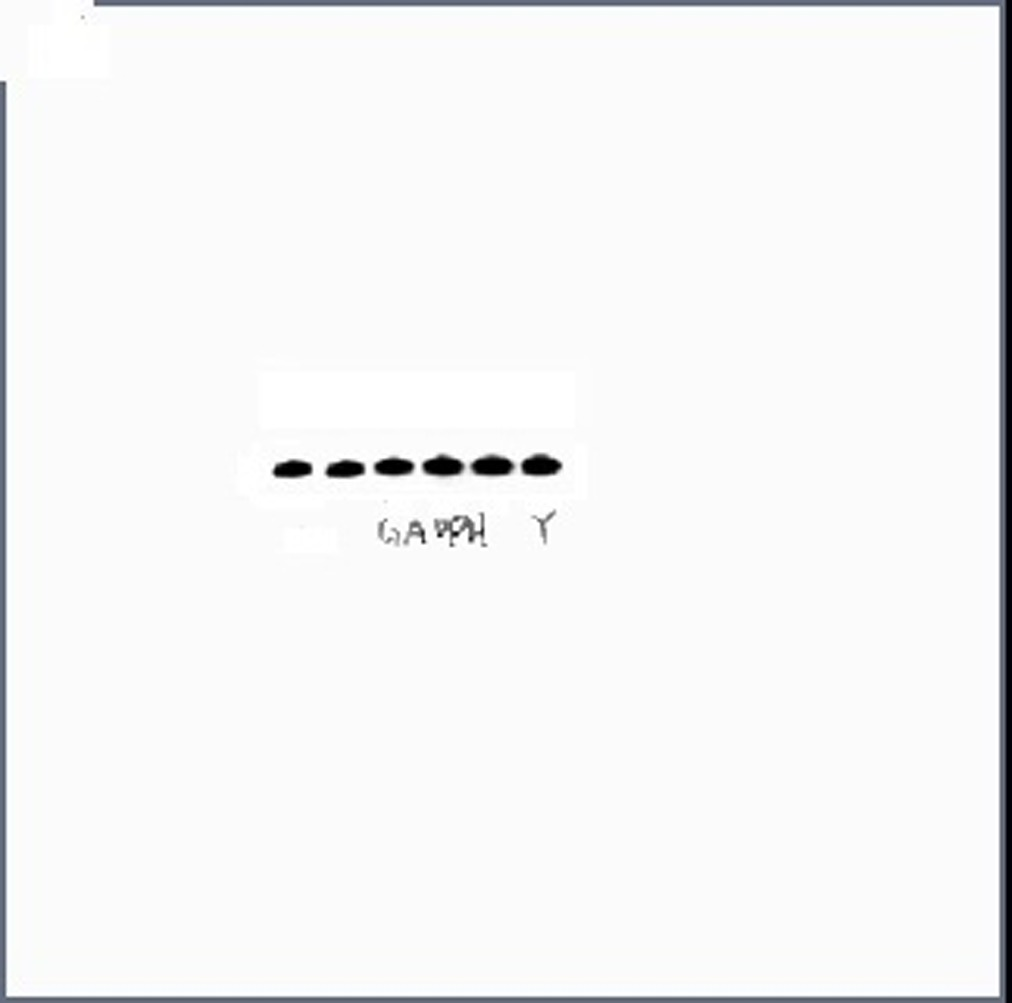

Supplement: S4 Fig — This is an original figure for GADPH as the internal reference of sGCβ1 by western-blot. (TIF) [file pone.0176777.s004.tif]

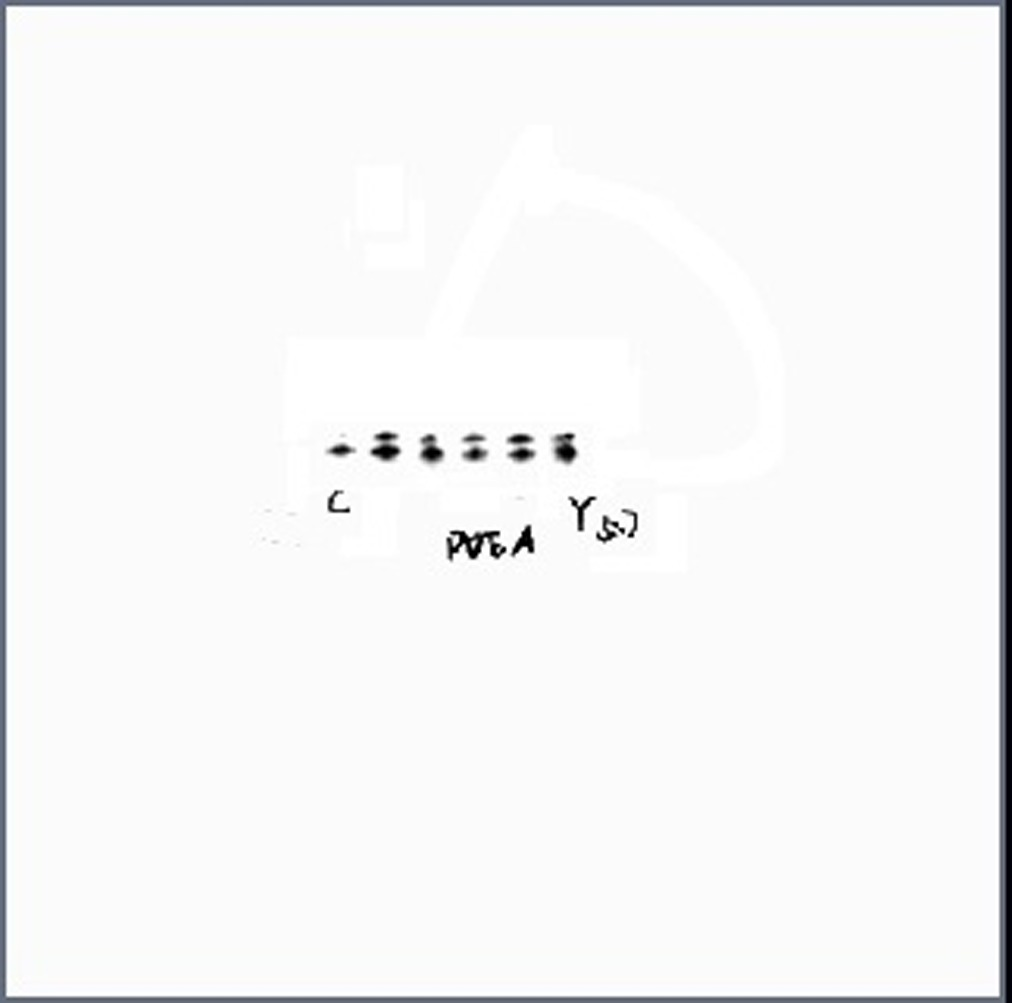

Supplement: S5 Fig — The original figure of PDE1A expression by western-blot. (TIF) [file pone.0176777.s005.tif]

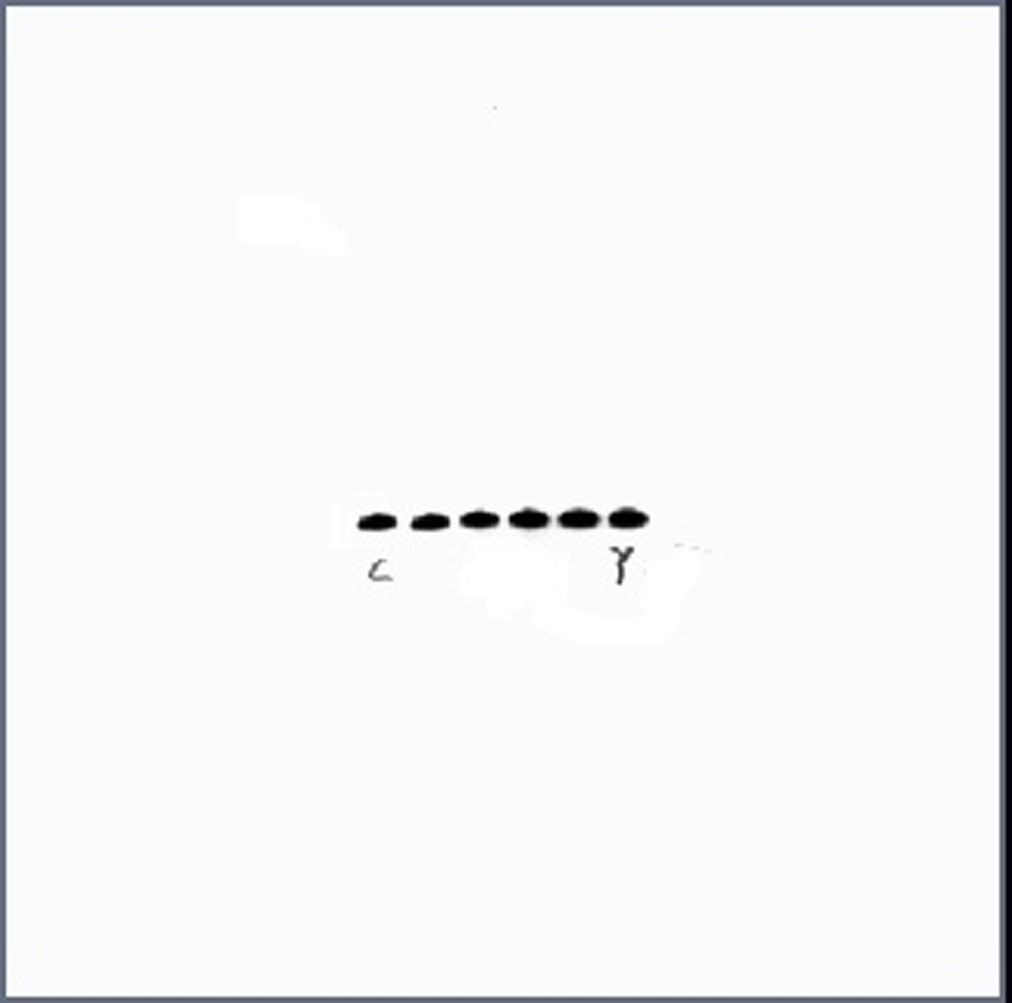

Supplement: S6 Fig — This is an original figure for GADPH as the internal reference of PDE1A by western-blot. (TIF) [file pone.0176777.s006.tif]

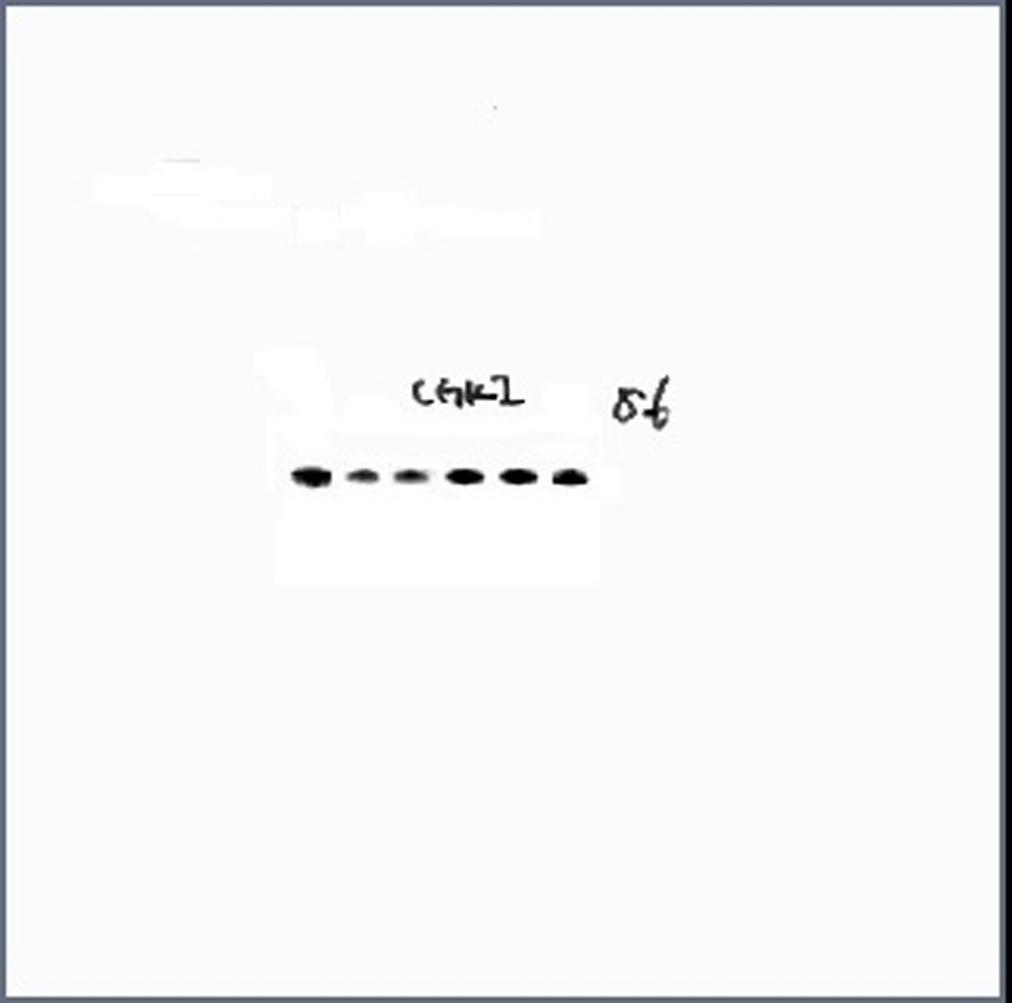

Supplement: S7 Fig — The original figure of cGK-I expression by western-blot. (TIF) [file pone.0176777.s007.tif]

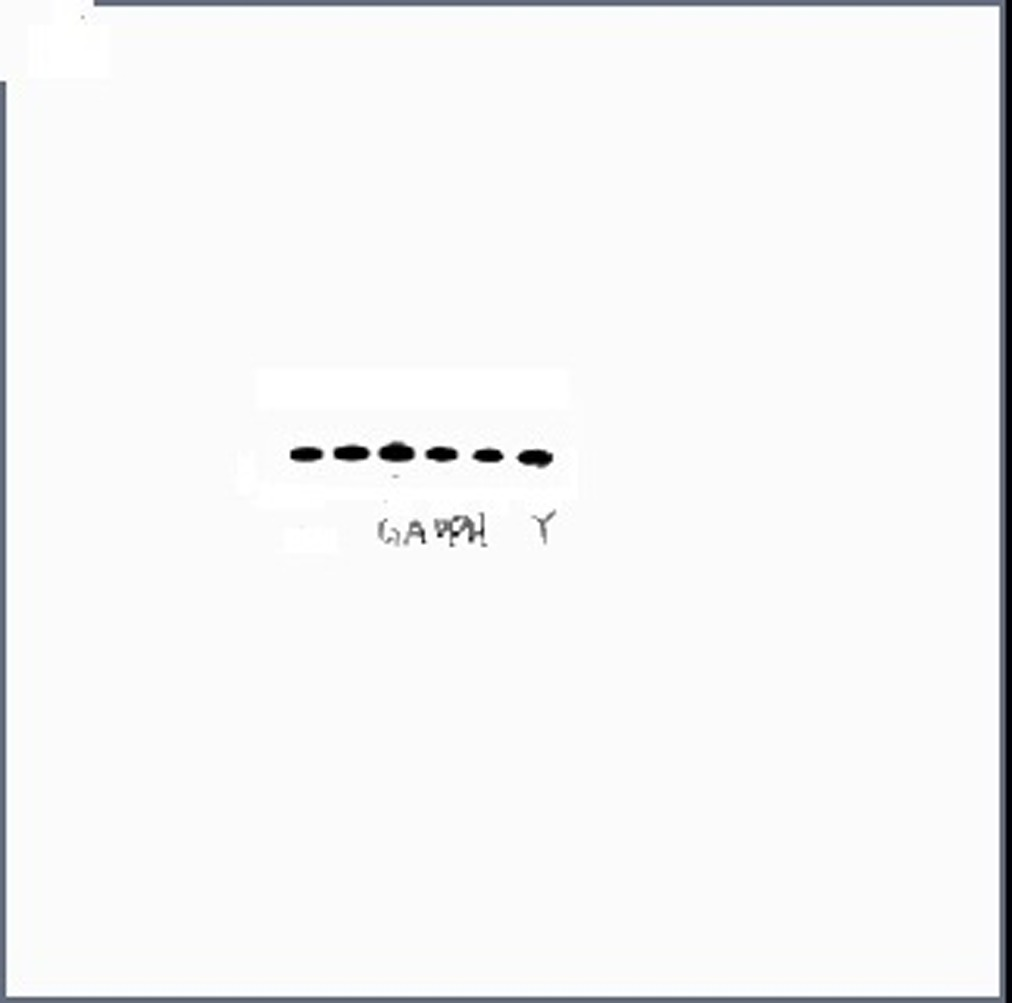

Supplement: S8 Fig — This is an original figure for GADPH as the internal reference of cGK-I by western-blot. (TIF) [file pone.0176777.s008.tif]

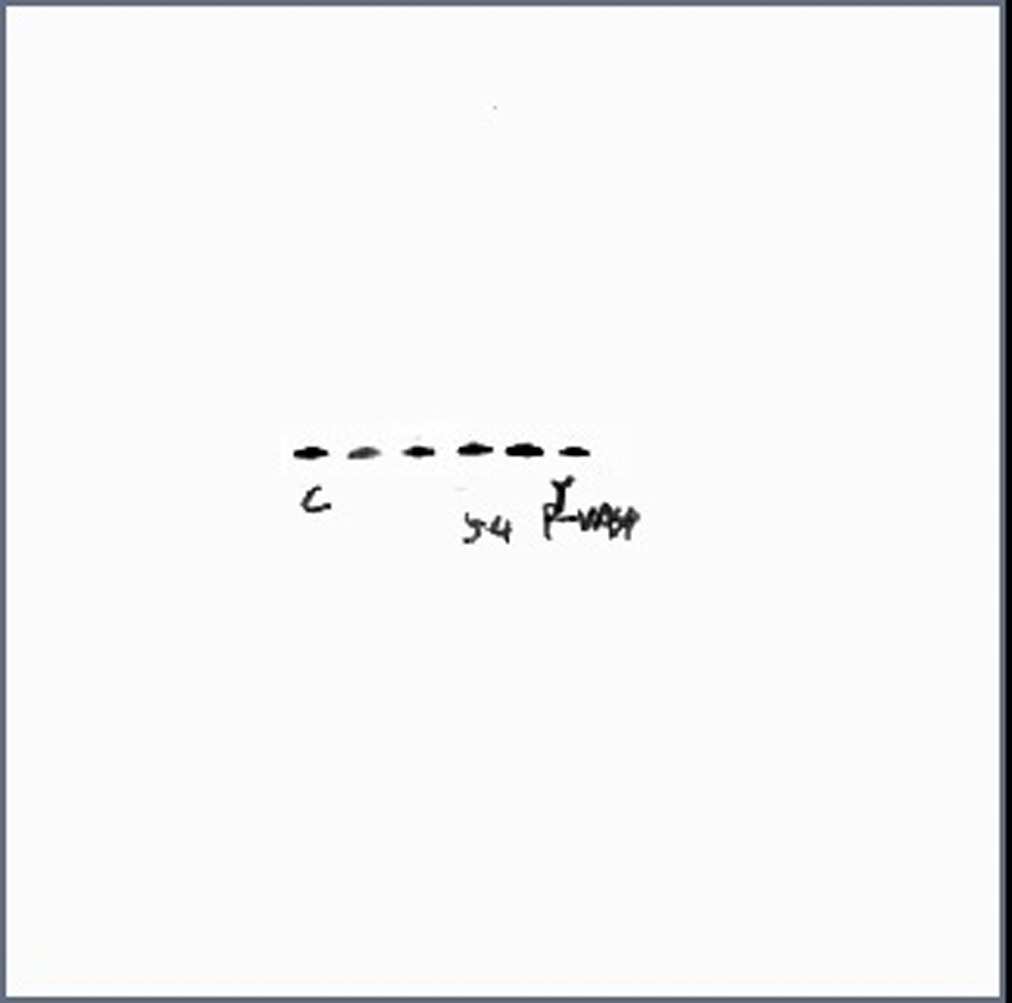

Supplement: S9 Fig — The original figure of P-VASP expression by western-blot. (TIF) [file pone.0176777.s009.tif]

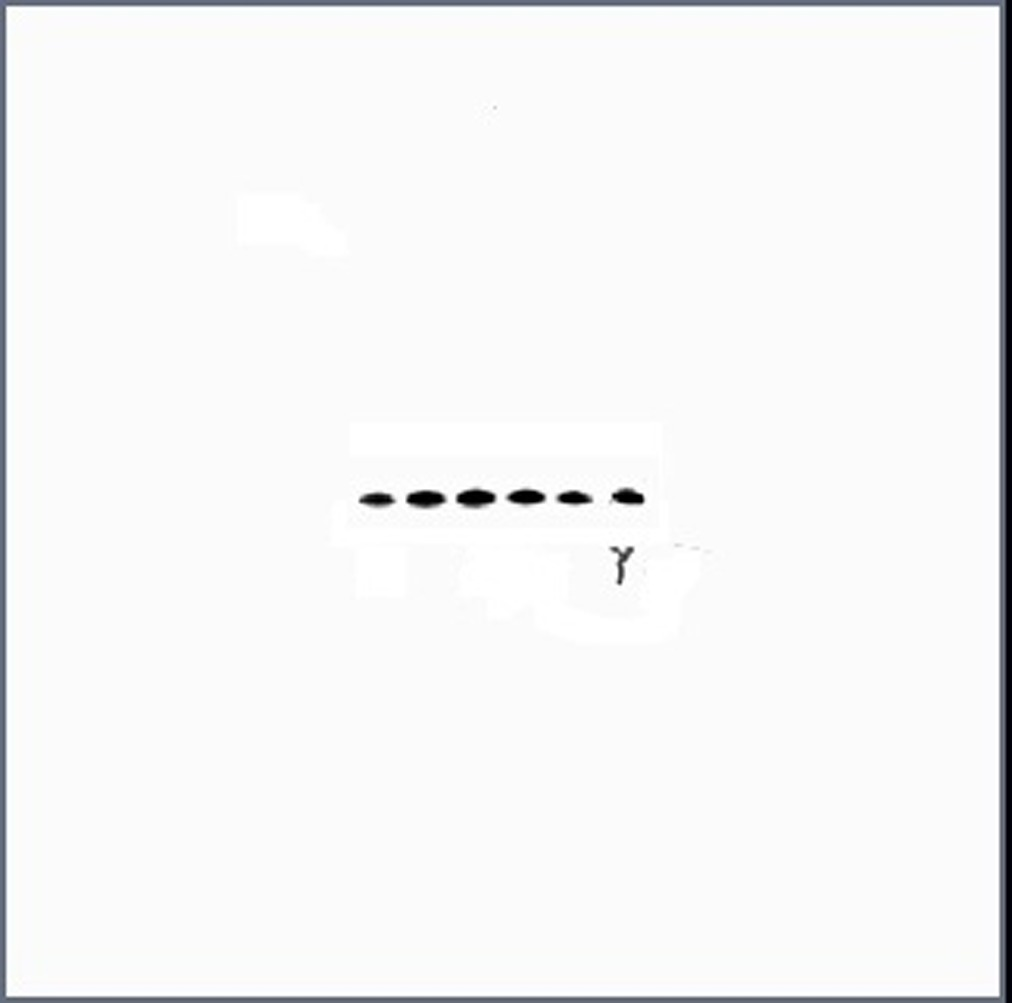

Supplement: S10 Fig — This is an original figure for GADPH as the internal reference of P-VASP by western-blot. (TIF) [file pone.0176777.s010.tif]
