# Supplementary material for: Effect of Shenmai injection on preventing the development of nitroglycerin-induced tolerance in rats
Source: PLoS One. 2017 Apr 28;12(4):e0176777. doi: 10.1371/journal.pone.0176777 (PMC5409518; doi:10.1371/journal.pone.0176777)
Supplement: S1 Text — Ultra-performance liquid chromatography/quadrupole time-of-flight mass spectrometry (UPLC/Q-TOF-MS) analysis of Shenmai injection. (DOC) [file pone.0176777.s011.doc]

In our previous study, we also have analyzed the chemical components of SMI using ultra-performance liquid chromatography/quadrupole time-of-flight mass spectrometry (UPLC/Q-TOF-MS). Because diﬀerent chemical components had better responses in diﬀerent modes, MS data were obtained in both positive ion mode (**Figure 1(A)**) and negative ion mode (**Figure 1(B)**). MS data in (+/−) ESI modes and the identification results for the constituents in Shenmai injection were presented in Table 1.


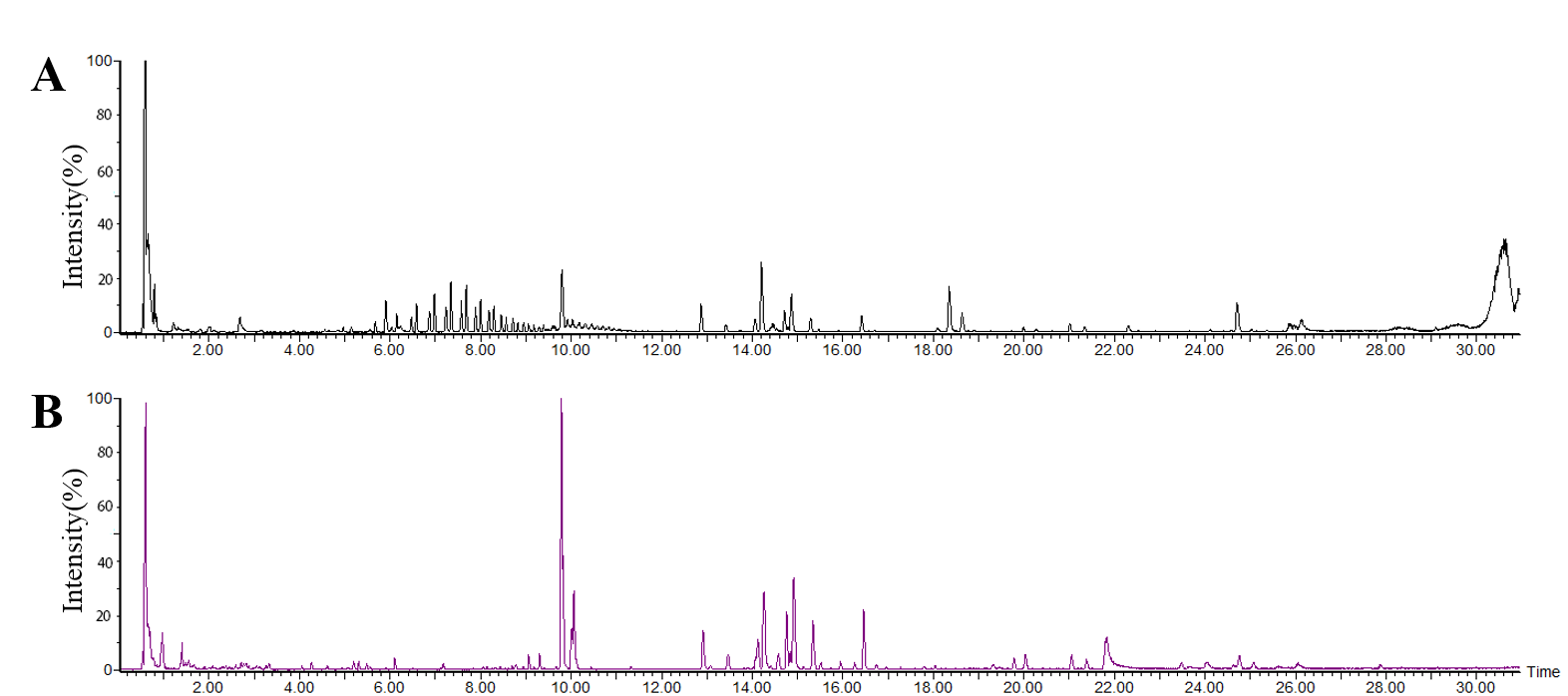


Figure 1: UPLC/Q-TOF-MS analysis of SMI. (A) Chromatograms of SMI in positive ion mode. (B) Chromatograms of SMI in negative ion mode.

Table 1: MS data in (+/−) ESI modes and the identification results for the constituents in SMI

| No. | Time  (min) | M/Z | Mode | MS-MS | Composition | Identiﬁcation |
| --- | --- | --- | --- | --- | --- | --- |
| 1 | 9.310 | 932.5327 | Neg/Pos | 931[M-H]^-^,637[M-H-Ara(p)-Glc]^-^,475[M-H-Ara(p)-2Glc]^-^ | C_47_H_80_O_8_ | Notoginsenoside R1 |
| 2 | 9.774 | 800.4920 | Neg/Pos | 799[M-H]^-^,637[M-H-Glc]^-^,475[M-H-2Glc]^-^ | C_42_H_72_O_14_ | Ginsenosides Rg1 |
| 3 | 9.823 | 946.4834 | Neg/Pos | 945[M-H]^-^,783[M-H-Glc]^-^,637[M-H-Glc-Rha]^-^ | C_48_H_82_O_18_ | Ginsenosides Re |
| 4 | 10.061 | 478.2230 | Neg/Pos | 477[M-H]^-^,459[M-H-H_2_O]^-^,315[M-H-Glc]^-^ | C_22_H_38_O_11_ | aplosy(1—6)  glucosamine bomeo |
| 5 | 12.916 | 800.4947 | Neg/Pos | 799[M-H]^-^,637[M-H-Glc]^-^,475[M-H-2Glc]^-^ | C_42_H_72_O_14_ | Ginsenosides Rf |
| 6 | 13.470 | 770.4785 | Neg/Pos | 769[M-H]^-^,637[M-H-Ara(p)]^-^,475[M-H-Ara(p)- Glc]^-^ | C_41_H_70_O_13_ | Notoginsenoside R2 |
| 7 | 14.104 | 784.4964 | Neg/Pos | 783[M-H]^-^,621[M-H-Glc]^-^,459[M-H-2Glc]^-^ | C_42_H_72_O_13_ | Ginsenosides Rg2 |
| 8 | 14.257 | 1108.5959 | Neg/Pos | 1107[M-H]^-^,945[M-H-Glc]^-^,783[M-H-2Glc]^-^ | C_54_H_92_O_23_ | Ginsenosides Rb1 |
| 9 | 14.760 | 1078.5916 | Neg/Pos | 1077[M-H]^-^,945[M-H-Ara(p)]^-^,915[M-H-Glc]^-^ | C_53_H_90_O_22_ | Ginsenosides Rc |
| 10 | 14.920 | 956.4962 | Neg/Pos | 955[M-H]^-^,937[M-H-H_2_O]^-^,793[M-H-Glc]^-^, | C_48_H_76_O_19_ | Ginsenosides Ro |
| 11 | 15.339 | 1078.5920 | Neg/Pos | 1077[M-H]^-^,945[M-H-Ara(p)]^-^,783[M-H- Ara(p)]^-^ | C_53_H_90_O_22_ | Ginsenosides Rb2 |
| 12 | 15.518 | 1078.5848 | Neg/Pos | 1077[M-H]^-^,915[M-H-Glc]^-^,783[M-H- Ara(p)]^-^ | C_53_H_90_O_22_ | Ginsenosides Rb3 |
| 13 | 16.460 | 946.5469 | Neg/Pos | 945[M-H]^-^,783[M-H- Glc]^-^,621[M-H-2Glc]^-^ | C_48_H_82_O_18_ | Ginsenosides Rd |
| 14 | 18.788 | 870.4498 | Neg | 869[M-H]^-^,851[M-H-H_2_O]^-^,737[M-H-Ara(p)]^-^ | C_44_H_70_O_17_ | Spirost-5-ene-3,17-diol,(3β,17αOH,25R)-from,  3-O-[α-L-rhamnopyranosyl-(1-2)-α-L-arabinofuranosyl-  (1-4)- β-D-glucopyranoside |
| 15 | 21.053 | 784.4973 | Neg/Pos | 783[M-H]^-^,621[M-H-2Glc]^-^,829[M-H+HCOOH]^-^ | C_42_H_72_O_13_ | iso-Ginsenosides Rg3 |
| 16 | 21.390 | 784.4973 | Neg/Pos | 783[M-H]^-^,621[M-H-2Glc]^-^,829[M-H+HCOOH]^-^ | C_42_H_72_O_13_ | Ginsenosides Rg3 |
| 17 | 25.071 | 766.4789 | Neg/Pos | 765[M-H]^-^,603[M-H- Glc]^-^,441[M-H-2Glc]^-^ | C_42_H_70_O_12_ | Ginsenosides Rk1 |
